# Effects of probucol on contrast-induced acute kidney injury in patients undergoing percutaneous coronary intervention

**DOI:** 10.1097/MD.0000000000016049

**Published:** 2019-06-21

**Authors:** Yong Wang, Yun Shi, Xuesheng Xu, Wenkun Ge, Shuo Yang, Chengzhi Lu

**Affiliations:** a First Center Clinic College of Tianjin Medical University, Tianjin; b Department of Cardiology; c Department of Hematology, The First People's Hospital of Shangqiu, Shangqiu, Henan; d Department of Cardiology, Tianjin First Center Hospital, Tianjin, China.

**Keywords:** contrast-induced acute kidney injury, preventive, probucol

## Abstract

**Objective:**

This study was performed to explore the effects of probucol on contrast-induced acute kidney injury (CIAKI) in patients with coronary heart disease undergoing percutaneous coronary intervention (PCI).

**Methods:**

In total, 220 patients undergoing PCI were randomly assigned to either the control group (hydration from 12 hours before to 12 hours after contrast administration; n = 110) or the probucol group (hydration plus probucol 500 mg twice daily 1 day before and 3 days after the operation; n = 110). The primary endpoint was the occurrence of serum creatinine (Scr)-based CIAKI, defined as an absolute increase in Scr by 0.5 mg/dl (44.2 μmol/L) or a relative 25% increase from baseline within 48 to 72 hours after exposure to contrast medium. The secondary outcomes were composite variations in Scr, blood urea nitrogen (BUN), creatinine clearance rate (Ccr) within 48 to 72 hours, and major adverse events during hospitalization or the 7-day follow-up period after PCI.

**Results:**

The overall incidence of Scr-based CIAKI was 7.3% (16/220): 5.5% (6/110) in the control group and 9.1% (10/110) in the probucol group (χ^2^ = 1.078, *P* = .298). There were no significant differences in the occurrence rate of major adverse events during hospitalization or the 7-day follow-up period after PCI between the groups. Multivariate logistic regression analysis showed that probucol was not an independent protective factor for CIAKI (odds ratio, 1.825; 95% confidence interval, 0.639–5.212; *P* = .261). However, hydration was an independent protective factor (odds ratio, 0.997; 95% confidence interval, 0.995–0.999; *P* = .004).

**Conclusion:**

Probucol cannot effectively reduce the incidence of CIAKI through its anti-inflammatory and antioxidative stress effects.

## 1 Introduction

Contrast-induced acute kidney injury (CIAKI) is a common complication after percutaneous coronary intervention (PCI), which is defined as an absolute increase in serum creatinine (Scr) by 0.5 mg/dl (44.2 μmol/L) or a relative increase of 25% from the baseline value within 72 hours after exposure to contrast medium.^[1]^ CIAKI is the third leading cause of acute kidney injury in hospitalized patients.^[2]^ CIAKI is associated with a prolonged duration of hospitalization as well as increased cardiovascular morbidity, kidney morbidity, and all-cause mortality; some patients even require dialysis.^[3,4]^ No effective treatment for CIAKI has been established, which emphasizes the need to identify effective measures to prevent CIAKI.

Probucol is a potential antioxidant drug that shows significant antioxidative stress and anti-inflammatory effects. It also helps to improve renal vascular endothelial function by reducing the endogenous nitric oxide synthase inhibitor concentration, increasing prostacyclin generation, inhibiting the expression of various adhesion molecules, and promoting the proliferation of endothelial cells while preventing their apoptosis due to oxidative injury.^[5,6]^ It is comprehensively used in clinical practice to prevent and treat atherosclerosis and diabetic nephropathy because of its strong antioxidative, anti-lipid peroxidation, and blood lipid-reducing effects. Some studies have shown that probucol plays a prophylactic role in the development of CIAKI.^[7]^ Most studies have shown that oxidative stress and inflammation play an important role in the pathogenesis of CIAKI.^[8,9]^ Thus, the antioxidative stress and anti-inflammatory effects of probucol may promote its use as a new medicine with which to prevent the occurrence of CIAKI. The purpose of this study was to investigate whether oral probucol can reduce the incidence of CIAKI in patients with coronary heart disease (CHD) undergoing PCI.

## 2 Methods

### 2.1 Study population

This study was a prospective, randomized, double-blind, controlled clinical trial. Patients with CHD scheduled for elective PCI in the Department of Cardiology of The First People's Hospital of Shangqiu from February 2017 to July 2018 were screened for eligibility. The inclusion criteria were an age of ≥18 years and the presence of CHD for which elective PCI was planned. The exclusion criteria were an allergy to contrast agents, acute or chronic infections, malignancies, emergency PCI, coagulopathy, exposure to contrast medium within 2 weeks of study entry, cardiogenic shock, severe cardiac insufficiency (left ventricular ejection fraction [LVEF] of <35%), electrolyte imbalance, severe renal insufficiency requiring short- or long-term dialysis, creatinine clearance rate (Ccr) of <30 ml/min, bronchial asthma, thyroid dysfunction, and use of kidney-toxic drugs or antioxidants (e.g., metformin, nonsteroidal anti-inflammatory drugs, ascorbic acid, or N-acetylcysteine) within 2 days of the procedure. The study was approved by the ethics committee of The First People's Hospital of Shangqiu, and all participants provided written informed consent.

### 2.2 Study protocol

All participants were randomly divided into either the control group (n = 110) or probucol group (n = 110) in a 1:1 ratio using computer-generated random numbers. Group allocation was carried out using undisclosed codes and recorded by nurses. Both the physicians and participants were blinded to the group endpoints and treatment interventions. Because treatment with saline during cardiovascular invasive procedures is recognized as the most effective strategy to prevent CIAKI, all selected patients were given intravenous sodium chloride at a rate of 1.0 ml^−1^ kg^−1^ h^−1^ from 12 hours before to 12 hours after the operation. Patients in the control group only received hydration. Patients in the probucol group received hydration and probucol (Qilu Pharmaceutical Co., Shandong, China) at 500 mg twice daily 1 day before and 3 days after the operation. Medication and saline were administered by the nurses. All patients underwent treatment with clopidogrel (loading dose of 300 mg followed by 75 mg/d) and aspirin (100 mg/d) for at least 12 months. In addition, angiotensin-converting enzyme inhibitors or angiotensin receptor antagonists, β-blockers, statins, calcium antagonists, diuretics, and other drugs were selected by the physicians according to the patient's condition and corresponding treatment guidelines. Drug delivery and hydration were performed by the nurses. The isosmotic contrast agent iodixanol injection (Jiangsu Hengrui Pharmaceutical Co., Ltd., Lianyungang, China) was used in all patients during PCI. None of the patients underwent antioxidant ascorbic acid or N-acetylcysteine therapy. PCI was performed by the same experienced interventional cardiologists.

### 2.3 Laboratory parameters

Blood samples were collected at baseline and at 48 and 72 hours after contrast exposure to measure the levels of blood urea nitrogen (BUN), Scr, beta-2 microglobulin (β2-MG), C-reactive protein (CRP), tumor necrosis factor α (TNF-α), malondialdehyde (MDA), superoxide dismutase (SOD), and glutathione peroxidase (GSH-PX). All blood tests were performed at the same hospital laboratory, and the laboratory staff was blinded to the study protocol and patients. The Ccr was calculated as follows: [140 − age] × weight (kg) / [0.818 × Scr (μmol/L)] (× 0.85 if female). The highest Scr level at 48 or 72 hours after PCI was used to diagnose CIAKI.

### 2.4 Study endpoints

The primary endpoint was the occurrence of Scr-based CIAKI, which was defined as an absolute increase in Scr by 0.5 mg/dl (44.2 μmol/L) or a relative increase of 25% from the baseline value within 48 to 72 hours after exposure to contrast medium. The secondary outcomes were (i) the changes in Scr and Ccr within 72 hours and (ii) major adverse events (including all-cause death, hypotension or severe decline in blood pressure, renal replacement therapy, internal bleeding, acute heart failure, postoperative emergency PCI or surgical coronary artery bypass after PCI, and cerebrovascular events) occurring during hospitalization and the 7-day follow-up period.

### 2.5 Statistical analysis

Normally distributed continuous variables (presented as mean ± standard deviation) were compared using Student's *t* test. Non-normally distributed continuous variables (presented as median and interquartile range) were analyzed using nonparametric tests. Categorical variables are reported as count and percentage, and the differences were analyzed with Pearson χ^2^ test or Fisher exact test. Multivariate logistic regression analyses were performed to analyze the protective factors for CIAKI after PCI. Odds ratios (ORs) and corresponding 95% confidence intervals (CIs) were calculated simultaneously. Based on previous research results, we speculated that the incidence of CIAKI after PCI would be 13% in the control group. We hypothesized that probucol could reduce the incidence of CIAKI to 10%. Accordingly, at least 92 patients in each group were required for a test with power set at 0.90 and a 2-sided *P* value of ≤.05. A 2-tailed *P* value of <.05 was considered statistically significant. All statistical analyses were carried out using SPSS software version 23.0 (IBM Corp., Armonk, NY).

## 3 Results

### 3.1 Baseline characteristics

**Figure 1:**
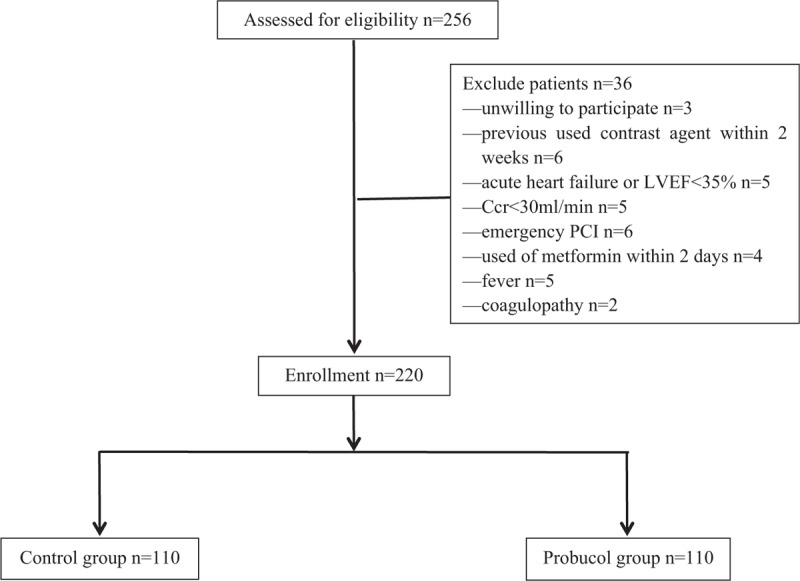
Flow diagram of the study selection process. Ccr = creatinine clearance rate, LVEF = left ventricular ejection fraction, PCI = percutaneous coronary intervention.

In total, 256 patients were initially enrolled. Of these patients, 36 were not included because they met the exclusion criteria (3 were unwilling to participate, 6 had used contrast agents within the past 2 weeks, 5 had acute heart failure or an LVEF of <35%, 5 had a Ccr of <30 ml/min, 6 had undergone emergency PCI, 4 had used metformin within the past 2 days, 5 had a fever, and 2 had coagulopathy). Finally, 220 patients with CHD were included and randomly divided into the control group (n = 110) and probucol group (n = 110) (Fig. 1).

The basic clinical, biochemical, and drug characteristics of the 220 participants are summarized in Table 1. There were no significant differences in the baseline characteristics between the 2 groups before PCI (*P* > .05).

**Table 1:**
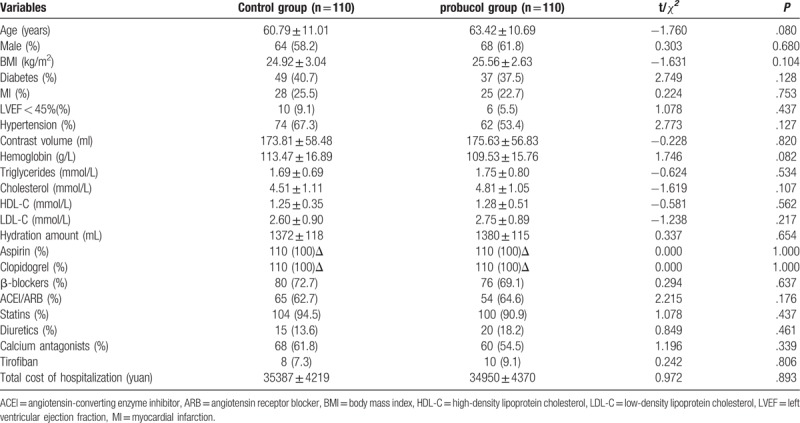
Comparisons of baseline characteristics between the 2 groups.

### 3.2 Changes in BUN, Scr, Ccr, β2-MG, CRP, TNF-α, MDA, GSH-PX, and SOD

The changes in BUN, Scr, Ccr, β2-MG, CRP, TNF-α, MDA, GSH-PX, and SOD were compared between the groups (Table 2). There were no statistically significant differences in the levels of Scr, BUN, Ccr, β2-MG, CRP, TNF-α, MDA, SOD, or GSH-PX between the groups before PCI (*P* > .05). There were also no statistically significant differences in the levels of Scr, BUN, Ccr, β2-MG, CRP, or TNF-α between the groups 72 hours after PCI (*P* > .05). The levels of CRP, SOD, and GSH-PX were significantly higher 72 hours after PCI than before PCI (*P* < .05). The level of MDA was significantly lower 72 hours after PCI than before PCI (*P* < .05). The probucol group had significantly lower levels of CRP, SOD, and GSH-PX 72 hours after PCI than did the control group (*P* < .05). The level of MDA 72 hours after PCI was significantly higher in the probucol group than control group (*P* < .05).

**Table 2:**
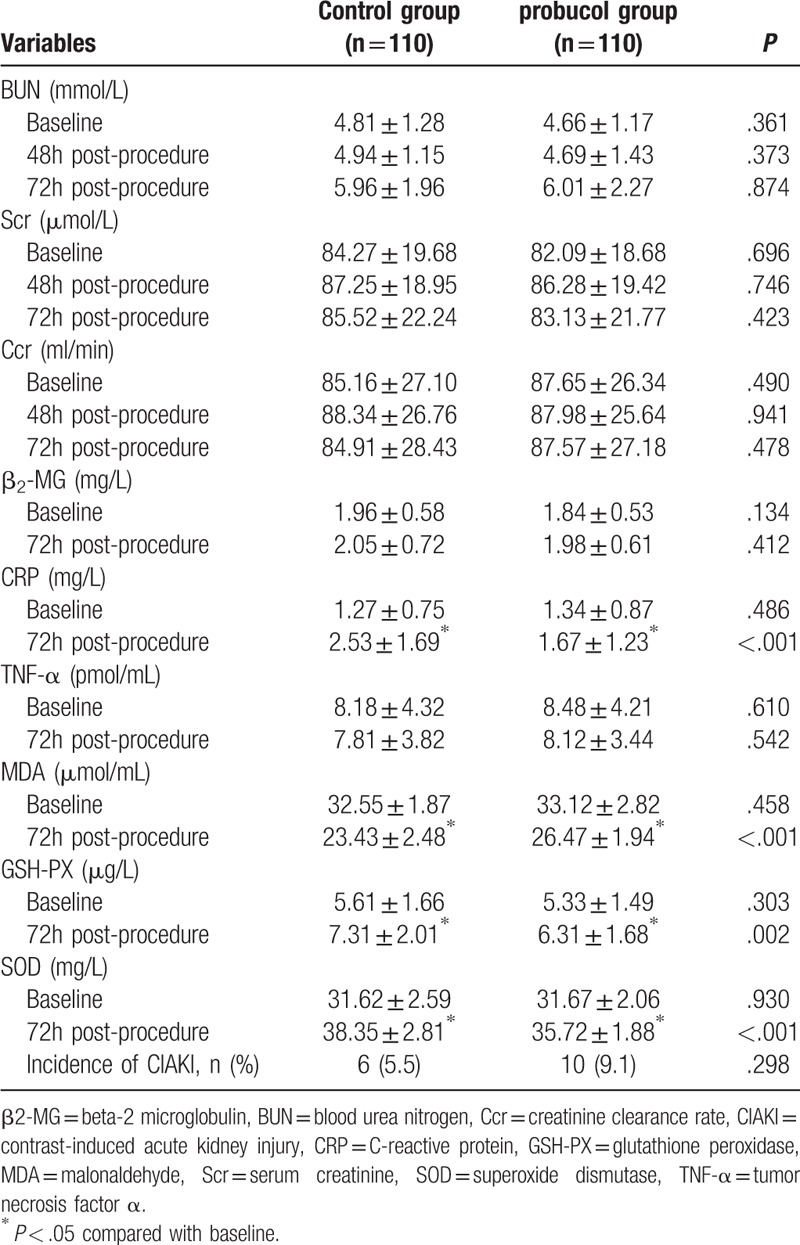
Changes in BUN, Scr, Ccr, β2-MG, CRP, TNF-α, MDA, GSH-PX, and SOD.

### 3.3 Incidence of CIAKI and multiple logistic regression analysis

The overall incidence of Scr-based CIAKI was 7.27% (16/220): 5.5% (6/110) in the control group and 9.1% (10/110) in the probucol group (χ^2^ = 1.078, *P* = .298). Multivariate logistic regression analysis was used to identify factors influencing CIAKI (myocardial infarction, LVEF of < 45%, contrast volume, angiotensin-converting enzyme inhibitors/angiotensin receptor blockers, diuretics, hypertension, diabetes, statins, age, and hydration amount). CIAKI was used as the dependent variable to exclude confounding factors. The multivariate logistic regression analysis showed that probucol was not an independent protective factor for CIAKI (OR = 1.825, 95% CI = 0.639–5.212, *P* = .261), while hydration was an independent protective factor for CIAKI (OR = 0.997, 95% CI = 0.995–0.999, *P* = .004) (Table 3).

**Table 3:**
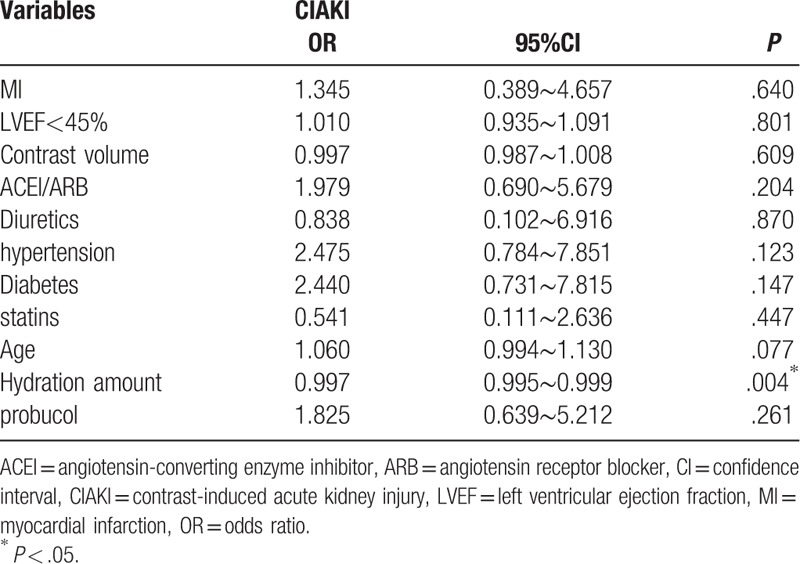
Multiple logistic regression analysis.

### 3.4 Major adverse clinical events during hospitalization and the 7-day follow-up period

There were no significant differences in major adverse clinical events between the 2 groups. During hospitalization and the 7-day follow-up period, 2 patients developed acute left heart failure and 1 patient developed cerebral hemorrhage in the control group, and 1 patient underwent emergency PCI for acute stent thrombosis and one patient developed gastrointestinal bleeding in the probucol group (2.7% vs 1.8%, respectively; χ^2^ = 0.205, *P* = .651). In the probucol group, three patients developed mild gastrointestinal discomfort such as abdominal distension and diarrhea, but no other adverse effects occurred. CIAKI occurred in 16 patients; of these, the Scr level returned to the normal range in 14 patients, and 2 patients were discharged from the hospital with an Scr level of 136 and 142 μmol/L, respectively.

## 4 Discussion

The present study showed that implementation of probucol at 500 mg twice daily 1 day before and 3 days after the operation alleviated the inflammatory reaction and oxidative stress; however, the incidence of CIAKI was not significantly different between the 2 groups (5.5% vs 9.1%, respectively; χ^2^ = 1.078, OR = 1.825, 95% CI = 0.639–5.212, *P* = .261).

With the widespread use of contrast medium, CIAKI has become a common cause of hospital-acquired acute kidney injury. CIAKI not only prolongs the hospitalization time and increases hospitalization expenses, but it also increases in-hospital mortality and the incidence of adverse events. A small proportion of patients with CIAKI may develop acute renal failure requiring temporary or chronic renal dialysis treatment.^[3,4]^ According to the current guidelines for the prevention of contrast-induced nephropathy, preventive methods include hydration before contrast agent exposure with normal saline solution or bicarbonates, reduction in the dosage of contrast medium, selection of iso-osmolar contrast medium, and discontinuation of nephrotoxic drugs 3 days prior to contrast medium use. However, even when these guidelines are followed, CIAKI remains a serious complication after contrast medium application. Hence, identification of effective ways to prevent the occurrence of CIAKI is urgently needed.

The pathogenesis of CIAKI is not completely clear. The main mechanisms of CIAKI after contrast administration may involve renal vasoconstriction, oxidative stress, an inflammatory response, and apoptosis.^[10]^ The role of oxidative stress and inflammation in the pathogenesis of CIAKI has attracted increasingly more attention. A possible treatment strategy for decreasing the risk of CIAKI is to use medication that maintains the balance between renal oxidative stress and inflammation.

Increasing numbers of experiments are demonstrating that probucol plays a prophylactic role in the development of CIAKI. In their multicenter randomized controlled trial, Fu et al^[11]^ found that prophylactic administration of probucol in the perioperative period of selective coronary intervention had a certain preventive effect against CIAKI. Yin et al^[12]^ showed that probucol can decrease the incidence of CIAKI in patients at high-risk of CIAKI with acute coronary syndrome undergoing primary or emergency angioplasty (4.2% vs 21.3%, *P* < .001), and the incidence of cystatin C-based CIAKI was significantly lower in the probucol than control group (29.2% vs 51.9%, respectively; *P* < .001). In addition, Li et al^[13]^ reported that atorvastatin combined with probucol decreased the serum uric acid level and incidence of CIAKI in patients undergoing PCI. However, whether probucol can effectively prevent the occurrence of CIAKI remains controversial. Lee et al^[14]^ showed that the use of probucol as an antioxidant during the perioperative period did not reduce the cell death caused by contrast agents. A recent meta-analysis showed that probucol did not effectively reduce the incidence of CIAKI (OR = 0.42, 95% CI = 0.15–0.91, *P* > .05).^[15]^ Interestingly, the results of our study showed that at 72 hours after PCI, the levels of Scr, BUN, Ccr, β2-MG, and TNF-α in the probucol group were not significantly different from those in the control group (*P* > .05). The incidence of CIAKI in the control group and probucol group was 5.5% and 9.1%, respectively, and the difference was not statistically significant. The results showed that the application of probucol alleviated the inflammatory reaction and oxidative stress but had no protective effect on renal function after PCI and did not effectively reduce the occurrence of CIAKI.

CRP is a sensitive index of the inflammatory response and is reportedly associated with CIAKI. Yuan et al^[16]^ and Gao et al^[17]^ reported that CRP is an independent predictor of CIAKI in that a higher CRP level is associated with a higher incidence of CIAKI. SOD and GSH-PX are free radical scavengers that are widely found in various tissues. They can scavenge the free radical O_2_^−^ (superoxide anion free radical) and have strong anti-inflammatory and antioxidant effects, and they can be used as an indicator of the oxidation capacity of the body. MDA is the end product of lipid peroxidation and is generated by the reaction between free radicals in vivo and unsaturated fatty acids under the cell membrane; thus, the MDA level reflects the body's antioxidative stress response.^[18]^ The SOD activity and the GSH-PX and MDA levels change due to generation of free radicals.^[19]^ Boyacioglu et al^[20]^ reported that the SOD and GSH-PX levels significantly decreased in the CIAKI group but significantly improved with L-carnitine therapy compared with the CIAKI group. Kongkham et al^[21]^ found that SOD enzyme activity decreased in the CIAKI group but significantly improved with alpha-tocopherol therapy. Previous studies of CIAKI and antioxidant agents have demonstrated an increase in MDA levels in the renal tissue in the CIAKI groups.^[20,21,22]^ In the present study, the levels of CRP, SOD, and GSH-PX were higher at 72 hours after PCI than preoperatively. The level of MDA was lower at 72 hours after PCI than preoperatively, and the probucol group had lower levels of CRP, SOD, and GSH-PX at 72 hours after PCI than the control group. At 72 hours after PCI, the MDA level in the probucol group was higher than that in the control group. These results show that an inflammatory reaction and oxidative stress occurred in the body after application of the contrast agent and that probucol has anti-inflammatory and antioxidative effects.

### 4.1 Study limitations

This study has 2 main limitations. First, it was a single-center randomized controlled clinical study, and the sample size may have been too small to reveal the difference in the incidence of CIAKI between the 2 groups. Second, the patients included in this study underwent elective PCI, and patients with severe cardiac and renal function were not included. All patients in this study were at low risk of CIAKI, resulting in a low incidence of CIAKI among the study population. This might also explain the lack of a significant difference in the incidence of CIAKI between the 2 groups.

## 5 Conclusions

The pathogenesis of CIAKI is complex. Probucol cannot effectively reduce the incidence of CIAKI through its antioxidative stress and anti-inflammatory effects. Whether probucol can effectively reduce the incidence of CIAKI requires further study in large-sample, multicenter, randomized, double-blind controlled trials.

## Acknowledgments

We thank all the researchers who participated in this work. We also thank Angela Morben, DVM, ELS, from Liwen Bianji, Edanz Editing China (www.liwenbianji.cn/ac), for editing the English text of a draft of this manuscript.

## Author contributions

**Conceptualization:** Yong Wang, Yun Shi.

**Data curation:** Xuesheng Xu, Wenkun Ge, Shuo Yang.

**Formal analysis:** Yun Shi.

**Project administration:** Xuesheng Xu.

**Supervision:** Chengzhi Lu.

**Writing – original draft:** Yong Wang.

**Writing – review & editing:** Chengzhi Lu.
